# Effect of Fertilization on the Performance of Adult *Pinus pinea* Trees

**DOI:** 10.3390/biology14020216

**Published:** 2025-02-19

**Authors:** Verónica Loewe-Muñoz, Claudia Bonomelli, Claudia Delard, Rodrigo Del Río, Monica Balzarini

**Affiliations:** 1 Instituto Forestal (INFOR), Santiago 4811230, Chile; cdelard@infor.cl; 2 Centro Nacional de Excelencia para la Industria de la Madera (CENAMAD), Pontificia Universidad Católica de Chile, Santiago 7820436, Chile; cbonomel@uc.cl (C.B.);; 3 Departamento de Fruticultura y Enología, Facultad de Agronomía e Ingeniería Forestal, Pontificia Universidad Católica de Chile, Santiago 7820436, Chile; 4 CONICET—Universidad Nacional de Córdoba, Av. Haya de la Torre s/n, Córdoba CC509, Argentina

**Keywords:** nutritional management, plantation management, silviculture, stone pine

## Abstract

Background: *Pinus pinea* L. (stone pine) produces pine nuts of high value. Its cultivation is carried out in forests and plantations, with intensive management techniques being studied to stimulate diameter growth, which is positively related to cone production. Aims: To evaluate the effect of fertilization in a 30-year-old plantation and to understand if adult trees respond to nutritional management. Methods: A trial with completely randomized block design was established with two treatments (fertilization/control) and three repetitions. The plantation, with a density of 204 trees/ha, is located in central Chile, on a sandy-loam soil with neutral pH, medium organic matter content, and a fertility condition that limits tree development. Fertilization considered the repeated application of macro (N, P, K, S, Mg) and micronutrients (B, Fe, and Zn). Periodic measurements of height, stem and crown diameter, and cone production were made up to age 36. Cone production was evaluated using mixed generalized linear models and growth variables using ANOVA (analysis of variance). Results: Significant effects of fertilization on DBH annual growth (35% higher than the control, *p* < 0.001) and in cone production (3 times higher, *p* < 0.0001) were found. Conclusions: Fertilization is a useful practice to improve the growth and cone productivity of the species.

## Simple Summary

*Pinus pinea* (stone pine) is a multipurpose Mediterranean species with interesting potential given its high growth and good adaptation in several countries, including Chile. Its greatest value lies in the production of cones, which contain pine nuts of high nutritional value. In general, its cultivation is carried out in unmanaged forests and plantations, with intensive management techniques being studied to stimulate diameter growth, which is positively related to fruit production. We evaluated the effect of fertilization in a 30-year-old plantation and found significant effects of fertilization on DBH annual growth (35% higher than the control) and in cone production (3 times higher). The results showed that fertilization is a useful practice to improve the productivity of the species.

## 1. Introduction

*Pinus pinea* Linnaeus., known as stone pine, is a multipurpose drought-resistant Mediterranean species whose greatest value lies in the production of cones, inside which is the pine nut, of high nutritional and culinary value. Indeed, pine nuts are among the most expensive nuts in the international market [1]. Thus, this species is being planted in several countries given its attractive socio-economic benefits, boosting rural development [2,3].

Some of these areas are located in the Southern Hemisphere, such as Chile, which has interesting potential given its good adaptation and development in a wide area of the territory [4], where over 5000 ha of stone pine plantations have been established in the last decade driven by the pine nut commercial potential.

In general, and unlike other nut-bearing trees, stone pine has not been domesticated, nor have varieties been used productively, with efforts to manage the species being recent and limited. In fact, species cultivation is carried out in unmanaged forest conditions, although semi-intensive or intensive management techniques are being studied to stimulate diameter growth, which is positively related to cone production [5]. Furthermore, growth variability [6] and differences in pine nut content [7,8] indicate the species’ sensitivity to soil features.

New approaches to forest management decisions are needed to adapt to climate change [9], which is increasing global temperatures and heat exposure, changing precipitation regimes, and increasing extreme weather events, alterations that most likely will reduce crop quality and yield [10]. Among these approaches, the use of climate-resilient crops—such as stone pine—along with undertaking actions to avoid the negative consequences of climate change are relevant. To guarantee good tree development, it is essential to ensure that nutrients are available in sufficient and balanced quantities; when this does not happen, it is necessary to implement practices that promote their availability and/or provide them through fertilization. Fertilization is a well-known technique to boost growth and fruiting in several arboreal species, including the pines *Pinus pinaster* Aiton [11], *Pinus edulis* Engelm., *Pinus monophyla* Torr. & Frém. [12], *Pinus taeda* Linnaeus [13], and *P. pinea* [14,15], and could contribute to the mitigation of climate change effects in semi-arid environments [16].

Fertilization effects on the other important pine nut producer species *Pinus koraiensis* Siebold & Zucc., as well as timber and cone production, have been investigated [17,18]. However, the effect of this practice on stone pine has been limitedly studied, including the species’ sensitivity to deficiency of the micronutrients boron [19] and iron [20] and the macronutrients nitrogen, phosphorus, calcium, and manganese [21]; calcium magnesium carbonate supply was addressed by Calama et al. [22] in Spain in an adult plantation, showing a positive response to fertilization in terms of pine nut production.

The goal of the study was to evaluate the effect of repeated fertilization on growth and cone production in a 30-year-old adult stone pine plantation located on the coast of central Chile. Our working hypothesis states that since the soil where the stone pine plantation was established has a low nutrient supply, by applying nutrients to the soil, growth and cone production are improved.

## 2. Materials and Methods

### 2.1. Study Site Characterization

The trial site is located in a coastal typically Mediterranean area of central Chile, with long, dry summers and short, intense winter rainfall, with the influence of the Pacific Ocean. Before trial establishment, a soil analysis was conducted following a systematic pattern in three randomly located zones; at each sampling point, soil from up to 60 cm depth was collected to build a composed sample. The soil is a sandy-loam with pH of 6.2 (neutral), medium organic matter content, and a mineral composition very low in phosphorus, boron, and sulfur; low in nitrogen and potassium; medium in copper, zinc, and iron; and high in manganese. Along with the shallow soil, this soil fertility condition limits tree development (Table 1).

### 2.2. Organism Studied

The studied organism corresponds to a 2 ha 30-year-old *Pinus pinea* L. plantation established to control the erosion product of repeated wheat cultivation. The management applied since plantation establishment includes two thinning events 20 and 30 years after planting, extracting 50% and 65% of trees, respectively, leaving a final density of 204 trees ha^−1^; and one pruning event at age 30.

The monitoring was carried out from 2013, when the trees were 30 years old, to 2019. In all trees, measurements of height, diameter-at-breast-height (DBH) at 130 cm above the ground, and crown diameter were taken in 2013, 2014, 2015, 2018, and 2019, and cones were counted in 2019, at age 36. Diameter measurements were performed to the nearest 0.1 cm with a caliper and tree height with a hypsometer to the nearest 0.1 m. Crown diameter was measured as the distance between the crown projections of living branches. Cones were visually counted from ground level.

At the beginning of the study, trees measured on average 5.7 m in height, with a 16.0 cm DBH (diameter-at-breast-height) and 3.6 m crown diameter.

### 2.3. Experimental Design

A trial with a completely randomized block design was established in 2013 with two treatments (fertilization/control) and three repetitions per treatment, and 25 trees per plot, totaling 75 monitored trees for each treatment. The trial was started to assess the effects of fertilization, with two treatments: control and fertilization (macronutrients (nitrogen, phosphorus, potassium, calcium, magnesium, sulfur) and micronutrients (boron, iron, zinc)). Fertilization was determined based on the soil nutrient contents selecting fertilizers available in the market. It was provided in partialized doses: in spring (when soil moisture and temperature necessary for growth are present) of years 2013, 2014, 2015, and 2018, consisting of Novatec N-max (1500 g plant^−1^), zinc sulfate (20 g plant^−1^), and borax (60 g plant^−1^); and in autumn (when soil moisture and temperature are still present, and nutrients are absorbed and reserved) [23] prior to the differentiation of female primordia that occurs in August in the area [24], of years 2014, 2015, 2016, and 2019, involving triple super phosphate (350 g plant^−1^) and carbamide (200 g plant^−1^). Nutrients supplied for each tree in spring are N (360 g), P (75 g), K (75 g), Mg (30 g), S (77 g), B (7 g), Fe (1 g), and Zn (5 g), and in autumn, P (161 g), Ca (49 g), and N (92 g).

### 2.4. Statistical Analyses

Cone production was evaluated using mixed generalized linear models with the Poisson family and growth variables using ANOVA (analysis of variance); in both cases, a Fisher Test was run (α = 0.05). Values are summarized in mean ± SE. Statistical analyses were performed using the software InfoStat (v.2024) [25] and its interface with the software R (v.3.6.3) (www.r-project.org).

## 3. Results

**Figure 1:**
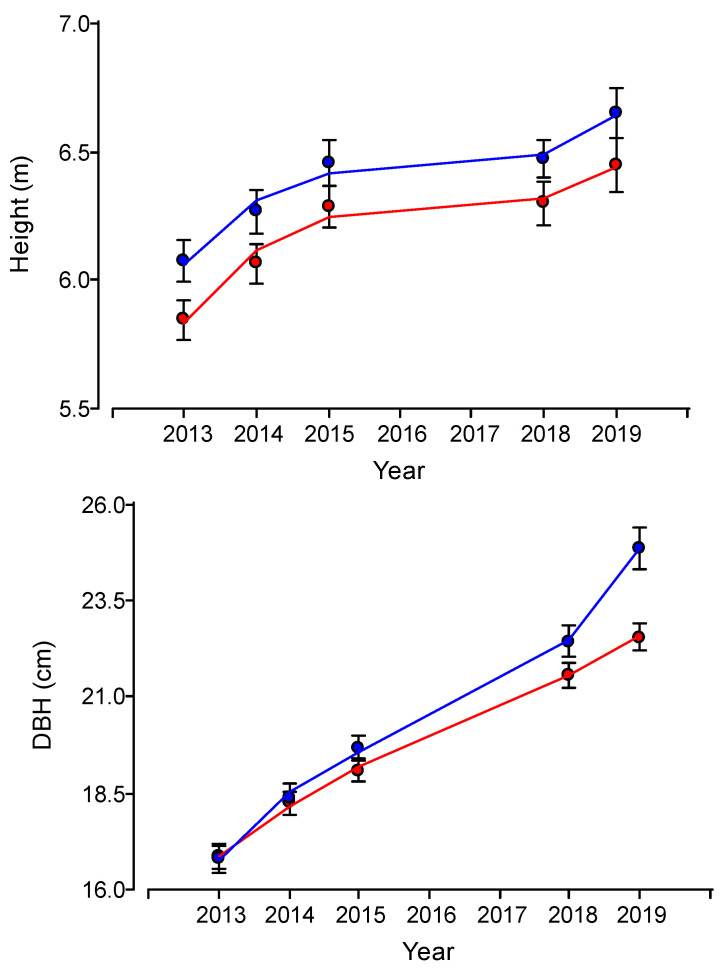

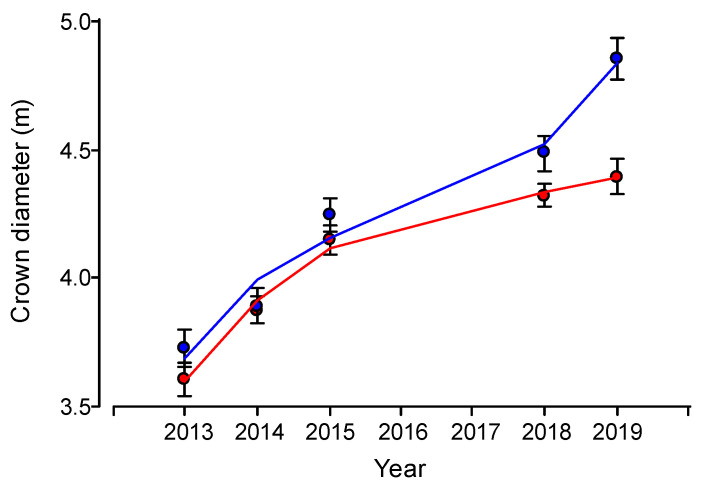
Stone pine tree size evolution from the experiment establishment in a plantation located in central Chile (blue: fertilization, red: control). Bars show standard errors.

**Figure 2:**
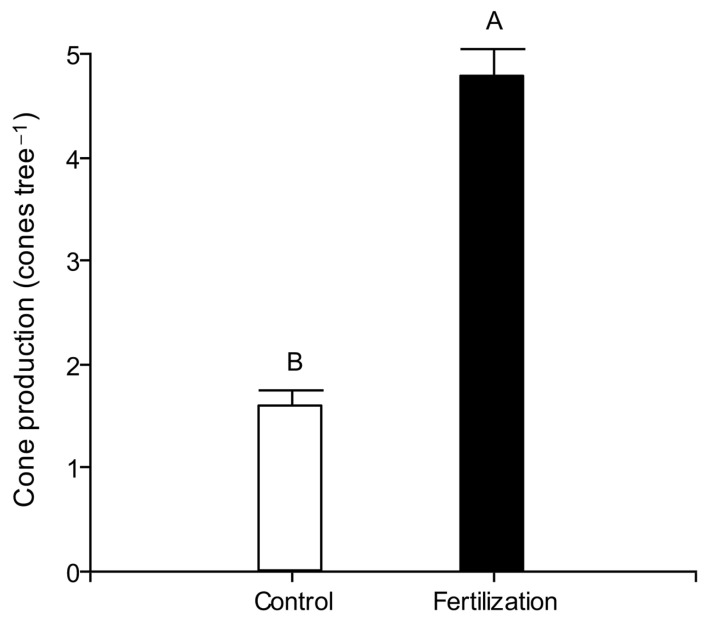
Cone production in 36-year-old stone pine trees in 2019, six years after the fertilization experiment started, in a xeric environment under drought. Bars show standard errors. Different letters indicate statistical differences among treatments at α = 0.05.

Vegetative growth rates in the five-year period (2014 to 2019) evaluated were 1.13 cm year^−1^ in DBH, 0.09 m year^−1^ in height, and 0.15 m year^−1^ in crown diameter. Results showed a significant effect of the treatment on DBH annual growth (*p* < 0.001) and in cone production (*p* < 0.0001), but no statistical differences were observed for height (*p* = 0.0503) or crown diameter (*p* = 0.0960). Fertilized trees had 35% higher DBH annual growth than untreated trees (control treatment) (1.34 vs. 0.99 cm year^−1^ respectively); even if not statistically significant (*p* > 0.05), fertilized trees also had a 25% higher height growth (0.10 vs. 0.08 m year^−1^ respectively) and 18% higher crown diameter growth than control trees (0.19 vs. 0.16 m year^−1^ respectively) (Figure 1). Regarding cone production, in 2019, fertilized trees had 3 times higher production than non-fertilized trees (4.8 vs. 1.6 cone tree^−1^ respectively) (Figure 2).

## 4. Discussion

Regarding vegetative growth, tree growth was reduced because the most limiting factor in the period in the studied site was water, since a severe continual mega-drought has affected the country, with rainfall drops of up to 40% [26], which affected both native forests and plantations.

In our study, fertilized trees’ average diameter growth increased by 35% in relation to non-fertilized trees, evidencing a positive effect of fertilization on *P. pinea* stem diameter growth, in agreement with experiences reported in Turkey by Kilci et al. [21]. Such a beneficial effect of nutrient supplementation was also reported in the same species on the coast of France [27] and in the central valley of Chile [28] in other pines such as *P. tropicalis* Morelet [29], *Pinus radiata* D. Don [30], and also in several *Eucalyptus* species [31]. However, we did not find significant effects of fertilizer supply on crown diameter, in disagreement with reports by Ravazi et al. [32], which could be explained by the reduced space available for tree crown expansion in the current 7 × 7 m spacing setting. Since a stronger negative effect on growth under warm conditions has been reported in plantations than in naturally regenerated stands [33], along with a negative trend for the resilience index in planted stands, fertilization could be studied as a management practice to help cope with climate change’s effects.

Cone production was very low because the most limiting factor in the studied period in the site was water, due to the prolonged drought. In spite of this, fertilized plots had a significantly three-times-higher mature cone production than non-fertilized ones, with this superior production being related to the highest biomass formation on trees, expressed through a higher growth rate in DBH and height than in control trees. A positive impact of the 2015 spring fertilization and 2016 fall fertilization in three-year-old cone number tree^−1^ was observed in the harvest of 2019, when cone production under fertilization was 3 times higher than in untreated plots, a difference that may have an economic impact at the stand level. We obtained a higher impact of fertilization than the one published by Kilci et al. [21], who found that a unique dose of macronutrient fertilization of 2360 g tree^−1^ resulted in 1.4 times superior cone production in comparison to untreated trees, which could be explained by the repeated and partialized supply used in this work (a total of 2130 g tree^−1^ year^−1^ repeated over four growth seasons). Our results are also higher that the ones reported by [34], who reported a 65% increase in cone production when applying fertigation. The effectiveness achieved from the tested fertilization could be explained by the repeated supplementation of minerals needed to support vegetative growth and to obtain high cone production, which, according to Marcelo et al. [35], include N, K, B, Mg, Fe, and Zn.

Considering the phenology of the species in the area [24], the periods in which fertilizers were supplied were appropriate, since autumn fertilization favored the development of female primordia, and spring fertilization favored flowering and growth. A positive effect of spring fertilization on *Pinus elliotti* Engelm. female cone induction was reported by Shoulders [36], and on *P. radiata* male cone production by Codesido and Merlo [37].

Our results indicate that if stone pine trees are nutritionally managed, even under drought, positive results are obtained for vegetative growth and cone production, representing a cropping alternative for arid zones.

Consequently, to increase the production potential, we recommend further studies carrying out soil analyses to determine deficits and supplies (environmental sustainability), as well as foliar analyses to monitor nutritional status to evidence absorption. Economic evaluations would also be necessary to assess the convenience of fertilizing under hydric deficit that reduces cone production.

Since Naves and Farinha [38] have indicated that stone pine cones from fertilized and irrigated trees show 15% higher damage from the Western conifer seed bug (*Leptoglossus occidentalis* Heidemann) than unmanaged trees, this effect should also be studied in trees subjected only to fertilization. Finally, considering that the effect of fertilization changes with different levels of water availability [39], studies including sites with lower and higher rainfall or irrigation are also recommended.

## 5. Conclusions

Fertilization increased the growth rate of adult stone pine trees, which translated into greater DBH. It also enhanced cone production, being a useful practice to improve the potential productivity of the species. This information is valuable for the sector, especially since stone pine is a species under domestication and there is little information on how to manage new plantations and old stands so that they produce more pine nuts.

## Figures and Tables

**Table 1:** Trial site characterization.

*Geographical location*	
Latitude	34°30′ S
Longitude	71°59′ W
Slope	15%
*Soil variables*	
Sand (%)	58
Lime (%)	38
Clay (%)	4
pH (H_2_O)	6.2
Salinity, EC (mmhos cm^−1^)	0.06
Organic matter (%)	1.7
*Climate variables* ^†^	
Average temperature (°C)	13.2
Average annual rainfall 1984–2012 (mm)	487
Average annual rainfall 2016 (mm)	174
Evapotranspiration (mm)	1200
Dry months per year	8.6

^†^ data obtained from DGA Pichilemu, Chile.

## Data Availability

Data will be made available on request.

## Glossary

Diameter-at-breast-height (DBH): diameter of the trunk of tree measured at 1.3 m height. Cone: the seed-bearing structure of *Pinus pinea* trees, inside which is the pine nut, of high nutritional and culinary value.

## References

[1] INC (2020). Statistical Review: Pine Nuts. Nutfruit.

[2] Schröder K., Khaldi A., Hasnaoui A. (2014). Analyse de la Chaîne de Valeur “Pignons de Pin” en Tunisie.

[3] Sattout E., Faour G., Carrasquinho I., Correia A., Mutke S. (2017). Insights on the value chain and management practices of stone pine forests in Lebanon. Mediterranean Pine Nuts from Forests and Plantations.

[4] Loewe V., Delard C., Balzarini M., Álvarez A., Navarro R. (2015). Impact of climate and management variables on stone pine (*Pinus pinea* L.) growing in Chile. Agric. For. Meteorol..

[5] Freire J.A., Rodrigues G.C., Tomé M. (2019). Climate Change Impacts on *Pinus pinea* L. Silvicultural System for Cone Production and Ways to Contour Those Impacts: A Review Complemented with Data from Permanent Plots. Forests.

[6] Court-Picon M., Gadbin-Henry C., Guibal F., Roux M. (2004). Dendrometry and morphometry of *Pinus pinea* L. in Lower Provence (France): Adaptability and variability of provenances. For. Ecol. Manag..

[7] Evaristo I., Batista D., Correia I., Correia P., Costa R. (2010). Chemical profiling of Portuguese *Pinus pinea* L. nuts. J. Sci. Food Agric..

[8] Vanhanen L., Savage G. (2013). Mineral analysis of Pine nuts (*Pinus* spp.) grown in New Zealand. Foods.

[9] Keenan R.J. (2015). Climate change impacts and adaptation in forest management: A review. Ann. For. Sci..

[10] Freitas T.R., Santos J.A., Silva A.P., Fraga H. (2023). Reviewing the Adverse Climate Change Impacts and Adaptation Measures on Almond Trees (*Prunus dulcis*). Agriculture.

[11] Zas R., Fernández-López J. (2005). Juvenile genetic parameters and genotypic stability of *Pinus pinaster* Ait. Open-pollinated families under different water and nutrient regimes. For. Sci..

[12] McLain R., Frazier P. (2008). Management Guidelines for Expanding Pinyon Nut Production in Colorado’s Pinyon-Juniper Woodlands.

[13] Maggard A.O., Will R.E., Wilson D.S., Meek C.R., Vogel J.G. (2016). Fertilization reduced stomatal conductance but not photosynthesis of *Pinus taeda* which compensated for lower water availability in regards to growth. For. Ecol. Manag..

[14] Loewe V., Álvarez A., Balzarini M., Delard C., Navarro-Cerrillo R. (2017). Mineral fertilization and irrigation effects on fruiting and growth in stone pine (*Pinus pinea* L.) crop. Fruits.

[15] Loewe-Muñoz V., Del Río Millar R., Delard Rodriguez C., Balzarini M. (2024). Effects of Fertilization on Radial Growth of *Pinus pinea* L. Explored Hourly Using Dendrometers. Ecol. Process..

[16] Loewe-Muñoz V., Cachinero-Vivar A.M., Camarero J.J., Del Río R., Delard C., Navarro-Cerrillo R.M. (2024). Dendrochronological Analysis of *Pinus pinea* L. in Central Chile and South Spain for Sustainable Forest Management. Biology.

[17] Shen H., Zhang P., Wu H., Wang Y., Li Y., Yin D. (2024). Biological Issues of Simultaneous Cultivation of Large-Diameter Bole and High-Yield Cones of Pinus koraiensis. Proceedings of the 26th IUFRO World Congress. Forest & Society Towards 2050.

[18] Zhao Y., Wang Z., Xu S., Li Y., He C. (2020). Nutrient Assimilation and Utilization in Korean Pine (*Pinus koraiensis*) Seedlings Exposed to Exponential Fertilization under Contrasting Spectra. Commun. Soil. Sci. Plant Anal..

[19] Bento J., Coutinho J. Boron deficiency in Stone pine. Proceedings of the Agropine 2011 International Meeting on Mediterranean Stone pine for Agroforestry.

[20] Malchi T., Shenker M. (2011). Iron Deficiency of Pinus pinea L.: Evaluation of Iron Uptake Mechanisms and Comparison of Different Genetic Lines.

[21] Kilci M., Akbin G., Sayman M., Özçankaya M. (2013). Determination of Effect of Fertilizing on Cone Yield of Stone Pine (Pinus pinea L.) in Kozak Province (Technical Bulletin).

[22] Calama R., Madrigal G., Candela J., Montero G. (2007). Effects of fertilization on the production of an edible forest fruit: Stone pine (*Pinus pinea* L.) nuts in the south-west of Andalusia. For. Syst..

[23] Li G., Wang J., Oliet J., Jacobs D. (2016). Combined pre-hardening and fall fertilization facilitates N storage and field performance of *Pinus tabulaeformis* seedlings. iForest—Biogeosci. For..

[24] Loewe V., Balzarini M., Álvarez A., Delard C., Navarro-Cerrillo R. (2016). Fruit productivity of Stone pine (*Pinus pinea* L.) along a climatic gradient in Chile. Agric. For. Meteorol..

[25] Di Rienzo J., Casanoves F., Balzarini M., Gonzalez L., Tablada M., Robledo C. (2024). InfoStat Version 2024. https://www.infostat.com.ar.

[26] Garreaud R.D., Boisier J.P., Rondanelli R., Montecinos A., Sepúlveda H.H., Veloso-Aguila D. (2019). The Central Chile Mega Drought (2010–2018): A climate dynamics perspective. Int. J. Climatol..

[27] Rapp M., Leclerc M., Lossaint P. (1979). The Nitrogen economy in a *Pinus pinea* L. stand. For. Ecol. Manag..

[28] Loewe-Muñoz V., Delard C., Del Río R., Balzarini M. (2020). Long-term effect of fertilization on stone pine growth and cone production. Ann. For. Sci..

[29] Ferrer A., Herrero G., Milián C., Aguirre B. (2004). Carencias nutrimentales en coníferas cubanas. I. *Pinus tropicalis* Morelet. Rev. For. Baracoa.

[30] Schlatter J., Gerding V. (1984). Deficiencia de Boro en Plantaciones de *Pinus radiata* D. Don en Chile II. Principales causas y corrección. Bosque.

[31] Bonomelli C., Suarez D. (1999). Fertilización del Eucalipto. Efecto sobre la acumulación de biomasa. Cienc. Investig. Agrar..

[32] Ravazi S., Azizi P., Rashidi R., Keivan F. (2006). The effect of NPK fertilizers on hand planting *Pinus pinea* L. in coastal areas of Caspian Sea. Iran. J. Nat. Resour. Res..

[33] Navarro-Cerrillo R.M., Rodriguez-Vallejo C., Silveiro E., Hortal A., Palacios-Rodríguez G., Duque-Lazo J., Camarero J.J. (2018). Cumulative Drought Stress Leads to a Loss of Growth Resilience and Explains Higher Mortality in Planted than in Naturally Regenerated *Pinus pinaster* Stands. Forests.

[34] Correia A.C., Farinha A., Silva J.E.P., Nunes A., Conceição N., da Encarnação Marcelo M., Sarmento A., Tomé M., Soares J., Fontes L. (2024). Fertirrigation in Grafted *Pinus pinea* L. Trees: Denser Crowns but No Effect on Cone Production or Masting Cycles. For. Ecol. Manag..

[35] da Encarnação Marcelo M., Correia A., Carvalho Partidário A., Gonçalves A.C., Alexandre C., Santos Silva C., Sempiterno C., Calouro F., Carrasquinho I., Silvestre J., UNAC—União da Floresta Mediterrânica (2022). A Fertilização do Pinheiro-Manso—Recomendações para uma Gestão Florest UNAC.

[36] Shoulders E. (1968). Fertilization increases longleaf and slash pine flower and cone crops in Louisiana. J. For..

[37] Codesido V., Merlo E. (2007). Inducción floral mediante aplicación de GA 4/7 y fertilización mineral en el huerto semillero de *Pinus radiata* D. Don de Sergude (Galicia). Investig. Agrar. Sist. Recur. For..

[38] Naves P., Farinha A. Nuevos conocimientos acerca de *Leptoglossus occidentalis* y *Dioryctria mendacella* en Portugal. Proceedings of the II Simposio del Pino Piñonero.

[39] Luo Y., D’Odorico P., Lee S.-C., Ma X., Migglivacca M., Peichl M., Stocker B., Gessler A. Divergent phenology response to nitrogen addition between a Mediterranean and a boreal forest. Proceedings of the EGU General Assembly 2023.

